# Withdrawal during outpatient low dose buprenorphine initiation in people who use fentanyl: a retrospective cohort study

**DOI:** 10.1186/s12954-024-00998-9

**Published:** 2024-04-09

**Authors:** Benjamin L. H. Jones, Michelle Geier, John Neuhaus, Phillip O. Coffin, Hannah R. Snyder, Christine S. Soran, Kelly R. Knight, Leslie W. Suen

**Affiliations:** 1 Medical Student Center, UCSF School of Medicine, 533 Parnassus Avenue, S-245, San Francisco, CA 94143 USA; 2 San Francisco Department of Public Health, 101 Grove Street, San Francisco, CA 94102 USA; 3 Department of Epidemiology and Biostatistics, University of California San Francisco, 550 16th Street, 2nd Floor, San Francisco, CA 94158 USA; 4 Department of Medicine, University of California San Francisco, 505 Parnassus Avenue, San Francisco, CA 94143 USA; 5 Department of Family and Community Medicine, University of California San Francisco, 995 Potrero Avenue, San Francisco, CA 94110 USA; 6 Division of General Internal Medicine, San Francisco General Hospital, 1001 Potrero Avenue, San Francisco, CA 94110 USA; 7 Division of Substance Abuse and Addiction Medicine, San Francisco General Hospital, University of California San Francisco, 1001 Potrero Avenue, San Francisco, CA 94110 USA; 8 Department of Humanities and Social Sciences, University of California San Francisco, 490 Illinois Street, 7th Floor, San Francisco, CA 94143 USA

**Keywords:** Opioids, Fentanyl, Opioid use disorder, Buprenorphine, Low dose buprenorphine initiation, Opioid agonist therapy, Opioid withdrawal, Precipitated withdrawal

## Abstract

**Background:**

Buprenorphine is an effective treatment for opioid use disorder (OUD); however, buprenorphine initiation can be complicated by withdrawal symptoms including precipitated withdrawal. There has been increasing interest in using low dose initiation (LDI) strategies to reduce this withdrawal risk. As there are limited data on withdrawal symptoms during LDI, we characterize withdrawal symptoms in people with daily fentanyl use who underwent initiation using these strategies as outpatients.

**Methods:**

We conducted a retrospective chart review of patients with OUD using daily fentanyl who were prescribed 7-day or 4-day LDI at 2 substance use disorder treatment clinics in San Francisco. Two addiction medicine experts assessed extracted chart documentation for withdrawal severity and precipitated withdrawal, defined as acute worsening of withdrawal symptoms immediately after taking buprenorphine. A third expert adjudicated disagreements. Data were analyzed using descriptive statistics.

**Results:**

There were 175 initiations in 126 patients. The mean age was 37 (SD 10 years). 71% were men, 26% women, and 2% non-binary. 21% identified as Black, 16% Latine, and 52% white. 60% were unstably housed and 75% had Medicaid insurance. Substance co-use included 74% who used amphetamines, 29% cocaine, 22% benzodiazepines, and 19% alcohol. Follow up was available for 118 (67%) initiations. There was deviation from protocol instructions in 22% of these initiations with follow up. 31% had any withdrawal, including 21% with mild symptoms, 8% moderate and 2% severe. Precipitated withdrawal occurred in 10 cases, or 8% of initiations with follow up. Of these, 7 had deviation from protocol instructions; thus, there were 3 cases with follow up (3%) in which precipitated withdrawal occurred without protocol deviation.

**Conclusions:**

Withdrawal was relatively common in our cohort but was mostly mild, and precipitated withdrawal was rare. Deviation from instructions, structural barriers, and varying fentanyl use characteristics may contribute to withdrawal. Clinicians should counsel patients who use fentanyl that mild withdrawal symptoms are likely during LDI, and there is still a low risk for precipitated withdrawal. Future studies should compare withdrawal across initiation types, seek ways to support patients in initiating buprenorphine, and qualitatively elicit patients’ withdrawal experiences.

**Supplementary Information:**

The online version contains supplementary material available at 10.1186/s12954-024-00998-9.

## Background

Opioid use disorder (OUD) and opioid overdose deaths have become critical public health issues in the U.S. and around the world [1, 2]. Fentanyl poses a significant problem in OUD treatment and overdose prevention because its high mu-opioid receptor binding affinity, potency, and risk for respiratory depression increase the risk of overdose death compared to heroin and other opioids [3]. The impacts of the opioid overdose crisis and fentanyl in the U.S. intersect deeply with structural issues including racism, poverty, and homelessness. People experiencing these and other forms of socioeconomic marginalization are more likely to experience OUD and to die of opioid overdose [4–6]. These circumstances have spurred efforts to develop effective OUD treatment strategies that meet patient preferences and needs.

Buprenorphine is a partial mu-opioid receptor agonist with high binding affinity that is an effective treatment for OUD, reducing all-cause mortality by over 50% [7–9]. Buprenorphine initiation has traditionally required a period of at least 8 h of opioid abstinence to prevent precipitated withdrawal, a rapid worsening of withdrawal symptoms from displacement of full-agonist opioids from the mu-opioid receptor [7, 10, 11]. Withdrawal symptoms including precipitated withdrawal can cause significant discomfort, may be associated with worse treatment outcomes, and can push patients to return to full-agonist opioid use and risk overdose [12–14]. People who use opioids describe precipitated withdrawal as a major concern with fentanyl use, and some describe it as a reason they avoid buprenorphine treatment or why they return to use [13, 15–18]. Multiple case reports describe patients with regular fentanyl use experiencing precipitated withdrawal even after waiting 8–24 h or more since their most recent use [19, 20]. It is hypothesized that with chronic use, fentanyl’s lipophilicity and low molecular weight lead to redistribution, accumulation, and prolonged release from the body’s peripheral tissues, facilitating an extended risk period for precipitated withdrawal [19, 21].

Little is known about individual pharmacokinetics in people chronically using high doses of fentanyl, and the exact incidence of precipitated withdrawal during outpatient buprenorphine initiation in people using fentanyl is unknown [22]. Self-reported data from 1679 OUD patients across the U.S. revealed odds of severe withdrawal during buprenorphine initiation were 5.2 times higher in people with fentanyl use in the past 24 hours compared to those with use in the past month [23]. Despite these early reports of severe and precipitated withdrawal during buprenorphine initiation, a recent prospective study of emergency department patients using fentanyl observed a precipitated withdrawal rate of only 0.76% [24]. This randomized control trial’s strong prospective study design suggests that the true rate of precipitated withdrawal may be much lower than has been reported elsewhere. However, this study included both patients receiving sublingual buprenorphine and extended-release injectable buprenorphine for initiation and took place in the emergency room where monitoring and administration of full-agonist prescription opioids is possible, making its findings difficult to apply to outpatient initiation using sublingual buprenorphine alone [24].

Among these uncertainties and rising fentanyl use, there have been calls for urgent exploration of new, effective and easier buprenorphine initiation strategies with reduced risk of withdrawal, including low dose initiation (LDI) [13]. LDI removes the need for opioid abstinence before initiation by preventing a sudden change in opioid signaling due to rapid displacement of full-agonist opioids from mu-opioid receptors [7, 25]. These strategies may reduce the incidence of precipitated withdrawal even in patients using fentanyl [26]. At the same time, LDI involves challenges including complex dosing regimens, need for ongoing use of full-agonist opioids, and splitting of buprenorphine tablets or films depending on dosing and available formulations [10, 26]. There are limited data regarding precipitated withdrawal, withdrawal severity, and symptoms in patients using fentanyl who undergo LDI as outpatients [19, 23, 27]. To fill this gap and inform clinical practice, we sought to characterize withdrawal symptoms experienced by a cohort of patients with daily fentanyl use who underwent outpatient LDI in an urban safety-net clinic system.

## Methods

### Study setting

We conducted a retrospective chart review of patients using daily fentanyl who received a prescription for buprenorphine LDI for OUD treatment at 2 clinics in San Francisco between May 2021 and November 2022. Both are substance use disorder treatment clinics collaboratively managed by the San Francisco Department of Public Health (SFDPH) and the University of California, San Francisco (UCSF). One primarily serves patients with various substance use disorders referred from Zuckerberg San Francisco General Hospital or outpatient SFDPH clinics. The other clinic primarily serves patients in the community who are interested in OUD treatment. Both clinics initiate and stabilize patients on buprenorphine before transitioning them to continuing care by a primary care or specialty mental health provider [26, 28]. They primarily serve patients who have public insurance (Medicare or Medicaid) or are uninsured [26, 28]. Buprenorphine prescriptions are dispensed by an SFDPH pharmacy specializing in mental health and substance use disorders [28]. This pharmacy provides additional behavioral health services including observed dosing, blister-packing of medications, naloxone and overdose education, and works with community organizations to provide harm reduction supplies [28].

At both clinics, patients interested in OUD treatment are seen by specialty addiction medicine physicians or nurse practitioners who gather substance use history including substances and routes of use, duration of use, complications and past overdoses, and prior treatment history. They also gather social, physical health, and mental health history, conduct a physical exam, and assess patients’ barriers and facilitators to buprenorphine treatment [26, 28]. Urine drug screens are offered at each visit, but may be declined by the patient.

### Buprenorphine initiation strategies

Providers work with patients to assess their preference for traditional initiation, 4-day rapid LDI (4-day), or 7-day standard LDI (7-day). Assessments include discussing patients’ desire to abstain from opioids for a sufficient period for traditional initiation, preferences and tolerance regarding withdrawal symptoms, and preference and ability to take medications up to 2–4 times daily for LDI protocols [26]. Patients then decide which method they prefer. Those who opt for LDI are counseled on the process and common concerns, including that they should continue full-agonist opioid (e.g. non-prescribed fentanyl) use during initiation as much as needed to avoid withdrawal symptoms, they may experience mild discomfort as they approach their full dose and adjust to a partial opioid agonist, and that discomfort may occur throughout initiation, including severe discomfort such as precipitated withdrawal [26]. Overdose prevention is discussed including naloxone use, using in the presence of others, and patients’ other self-determined practices to reduce overdose risk [26]. Patients are encouraged to contact their clinician if they have concerns or significant discomfort including precipitated withdrawal [26, 28].

The dosing schedules for the 4-day and 7-day LDI protocols are described in Table 1. Patients are instructed to follow up with their provider after taking their 12 mg buprenorphine dose on the final day of their protocol, but are encouraged to follow up earlier as needed [26]. Adjunctive medications including ondansetron, clonidine, ibuprofen, hydroxyzine, and loperamide are offered and may be prescribed as well [28]. Patients receive their buprenorphine from the SFDPH specialty behavioral health pharmacy along with medication counseling by behavioral health pharmacists. Both LDI protocols utilize sublingual buprenorphine or buprenorphine-naloxone tablets in blister packs to promote clarity during dose uptitration [26].

**Table 1:** Rapid low dose (4-day) and standard low dose (7-day) initiation protocol dosing schedules

Day of protocol	Rapid (4-day) protocol	Standard (7-day) protocol
1	0.5 mg every 6 h	0.5 mg once
2	1 mg every 6 h	0.5 mg in the morning and evening
3	2 mg every 6 h	0.5 mg in the morning, then 1 mg in the afternoon and evening
4	12 mg in the morning, then follow up with provider	2 mg in the morning and evening
5		3 mg in the morning and evening
6		4 mg in the morning and evening
7		12 mg in the morning, then follow up with provider

### Data collection and measures

We abstracted all buprenorphine initiations that were picked up from the SFDPH specialty behavioral health pharmacy from May 2021 to November 2022 from the electronic health record. Patients were excluded if they were undergoing LDI to transition to buprenorphine from treatment with methadone, were prescribed a non-standardized LDI protocol, did not report regular fentanyl use or reported no fentanyl use in the past month, had buprenorphine positive urine drug screens at initiation, or had insufficient chart documentation during intake. Further, while precipitated withdrawal during traditional initiations is generally quite discrete and delineated, it is often vaguer and more ill-defined during LDI. We felt that direct comparison between traditional initiations and LDI was not possible in our sample and therefore opted not to include traditional initiations in our study.

Two researchers (BLHJ and LWS) used a standardized chart abstraction protocol to review all clinic notes associated with buprenorphine initiation and collect data on patient demographics, psychiatric and medical comorbidities, substance use and treatment history, prescribed buprenorphine initiation protocol, protocol deviation, and withdrawal symptoms. Deviation from protocol was defined as any significant departure from prescribed protocol instructions such as skipping doses, taking higher doses ahead of schedule, further splitting tablets into smaller doses, or intentionally prolonging the initiation process beyond the 4- or 7-day period. Patients were noted as having any withdrawal if chart documentation explicitly mentioned that they experienced any symptoms during initiation that were possibly related to withdrawal. As providers at follow up visits regularly screen for withdrawal and appropriately document reported symptoms, we assumed that if no withdrawal symptoms were mentioned in follow up notes, then patients did not experience any during their initiation.

Subjective (patient history) and objective (physical exam) descriptions of withdrawal symptoms were extracted from the follow up notes for all patients for whom withdrawal was documented. Extracted descriptions were de-identified and any references to the prescribed protocol were redacted. We used extracted chart documentation on withdrawal to identify types of withdrawal symptoms experienced (e.g., agitation, anxiety, etc.), as adapted from the Subjective Opiate Withdrawal Scale [29].

Two expert addiction medicine physicians (HRS and CSS), blinded to protocol type, then assessed extracted chart documentation for the occurrence of precipitated withdrawal and severity of withdrawal. They were instructed to use their clinical judgment to determine whether withdrawal symptoms were mild, moderate, or severe, and whether there was any concern for precipitated withdrawal from buprenorphine. Though some studies have used changes in Clinical Opiate Withdrawal Scale (COWS) [30] scores to describe precipitated withdrawal, there is no standardized operational definition [11, 22, 24]. We were unable to use objective scoring systems, so we defined precipitated withdrawal as documentation of the patient experiencing acute worsening of withdrawal symptoms immediately after taking buprenorphine [19]. Agreement between experts was calculated using Cohen’s kappa. In cases of disagreement, a third addiction medicine expert (LWS) adjudicated.

Data were analyzed using descriptive statistics in Stata version 18.0 [31]. All study procedures were approved by the UCSF Institutional Review Board (IRB #21-33732).

## Results

There were 126 unique patients who underwent a total of 175 initiation attempts. Patient characteristics are presented in Table 2. The mean age was 37 years (SD 10 years) and the majority were men (71%), while 26% were women and 2% were non-binary. 21% identified as Black or African American, 16% as Latine, 52% as white, and 10% as another race. The majority of patients were publicly insured, with 75% having Medicaid and 6% Medicare, while 6% had no insurance. 37% were stably housed, 35% were transitionally housed (living in a shelter, hotel, or with family or friends), and 25% were unsheltered (living in a car or on the street).

**Table 2:** Patient characteristics, by protocol

	Total	4-Day	7-Day
	*N* = 126	*N* = 54	*N* = 72
**Age**	37(10)	36(9)	38(11)
**Gender**			
Woman	33 (26%)	16 (30%)	17 (24%)
Man	90 (71%)	38 (70%)	52 (72%)
Non-binary	3 (2%)	0 (0%)	3 (4%)
**Race/ethnicity**			
Black or African American	26 (21%)	10 (19%)	16 (22%)
Latine	20 (16%)	7 (13%)	13 (18%)
White	66 (52%)	31 (57%)	35 (49%)
Other	13 (10%)	6 (11%)	7 (10%)
Unknown or not available	1 (1%)	0 (0%)	1 (1%)
**Primary insurance**			
Medicaid	94 (75%)	45 (83%)	49 (68%)
Medicare	8 (6%)	1 (2%)	7 (10%)
Uninsured	8 (6%)	4 (7%)	4 (6%)
Other	16 (13%)	4 (7%)	12 (17%)
**Housing status**			
Stably housed	47 (37%)	18 (33%)	29 (40%)
Transitionally or temporarily housed	44 (35%)	17 (31%)	27 (38%)
Unsheltered	32 (25%)	17 (31%)	15 (21%)
Unknown or not available	3 (2%)	2 (4%)	1 (1%)
**Medical and psychiatric history**			
Chronic pain	33 (26%)	18 (33%)	15 (21%)
Depression	53 (42%)	24 (44%)	29 (40%)
Bipolar disorder	21 (17%)	8 (15%)	13 (18%)
Anxiety disorder (GAD, OCD, PTSD)	56 (44%)	21 (39%)	35 (49%)
ADD/ADHD	17 (13%)	5 (9%)	12 (17%)
Psychosis	25 (20%)	6 (11%)	19 (26%)
**Substance co-use in the past 3 months**			
Alcohol	24 (19%)	9 (17%)	15 (21%)
Benzodiazepine	28 (22%)	12 (22%)	16 (22%)
Amphetamines	93 (74%)	35 (65%)	58 (81%)
Cocaine	37 (29%)	22 (41%)	15 (21%)
**Opioid use in the past 3 months in addition to fentanyl**			
Opioid pills	2 (2%)	0 (0%)	2 (3%)
Heroin	31 (25%)	12 (22%)	19 (26%)
**Primary route of opioid use in past 3 months**			
Injection (IV/IM)	13 (10%)	6 (11%)	7 (10%)
Inhaled (smoked)	99 (79%)	41 (76%)	58 (81%)
Intranasal (snorted)	5 (4%)	2 (4%)	3 (4%)
Unknown or not available	9 (7%)	5 (9%)	4 (6%)
**History of drug overdose**	83 (66%)	38 (70%)	45 (62%)
**Prior buprenorphine treatment**	87 (71%)	34 (64%)	53 (76%)

**Table 3:** Clinician-validated withdrawal severity and occurrence of precipitated withdrawal among initiation attempts, by protocol

		Total	4-Day	7-Day
		*N* = 118	*N* = 54	*N* = 64
**Any withdrawal**		37 (31%)	17 (31%)	20 (31%)
**Severity of withdrawal**				
None		81 (69%)	37 (69%)	44 (69%)
Mild		25 (21%)	10 (19%)	15 (23%)
Moderate		10 (8%)	6 (11%)	4 (6%)
Severe		2 (2%)	1 (2%)	1 (2%)
**Precipitated withdrawal**				
Not enough information		22 (19%)	6 (11%)	16 (25%)
No		86 (73%)	42 (78%)	44 (69%)
Yes		10 (8%)	6 (11%)	4 (6%)
	**Deviated from protocol?**			
	No	3 (3%)	1 (2%)	2 (3%)
	Yes	7 (6%)	5 (9%)	2 (3%)

Psychiatric comorbidities included 42% with a history of major depressive disorder, 17% bipolar disorder, 44% anxiety disorder (generalized anxiety disorder, obsessive compulsive disorder, post-traumatic stress disorder), 13% attention deficit disorder or attention deficit hyperactivity disorder, and 20% psychotic disorder. Substance co-use in the past 3 months included 74% reporting amphetamine use, 29% cocaine, 19% alcohol, and 22% benzodiazepines. In addition to fentanyl, 25% reported heroin use in the past 3 months and 2% reported use of other opioid pills. The most common primary route of use was inhalation/smoking, reported by 79% of patients, while 10% reported primarily injecting, and 4% intranasal use/snorting. 66% had a history of drug overdose, while 71% had a history of prior treatment with buprenorphine.

We had follow up data for 118 (67%) initiation attempts. There was known deviation from initiation protocol instructions in 22% of these initiations. The frequencies of specific withdrawal symptoms by protocol are available as a supplement (Supplemental Table 1). The most common symptoms were restlessness, anxiety, and nausea. The supplement also displays adjunctive medications for withdrawal symptoms prescribed at initiation (Supplemental Table 2), though we do not have data on whether these medications were used by patients during initiation.

Interrater agreement between addiction medicine experts was 61% (kappa = 0.37) for withdrawal severity and 84% (kappa = 0.72) regarding possible precipitated withdrawal. Among the 118 initiations with follow up data, there were 31% in which patients experienced any withdrawal symptoms, including 21% with mild symptoms, 8% moderate, and 2% severe. The proportions of each severity category were similar in the 4-day and 7-day protocols.

Of all 118 initiations with follow up data, 19% lacked sufficient documentation to assess for the presence of precipitated withdrawal, meaning that chart documentation lacked information about timing of withdrawal symptoms necessary to know whether withdrawal symptoms happened immediately after buprenorphine administration. Precipitated withdrawal was seen in 10 attempts (8% of all those with follow up data). Six cases were in 4-day initiations (11% of 4-day initiations with follow up data) and 4 cases were in 7-day initiations (6% of 7-day initiations with follow up data). In these cases of precipitated withdrawal, clinician experts deemed two cases as having severe withdrawal and the rest as moderate severity. There was known deviation from protocol instructions in 7 of the 10 cases of precipitated withdrawal. Therefore, of 118 patients with follow up data, 3 (3%) had precipitated withdrawal despite having no known deviation from protocol instructions.

Types of protocol deviation included taking higher doses of buprenorphine ahead of schedule (*N* = 3), skipping or missing doses before taking a higher dose (*N* = 2), and becoming confused or forgetting protocol instructions (*N* = 2). Among the 2 patients who reported becoming confused or forgetting instructions, one patient forgot the instructions for their 4-day protocol and decided to take one dose per day until all tablets were used before finally taking their 12 mg dose and experiencing withdrawal. The other stated to their provider that the 7-day protocol “can be confusing,” and described following the protocol until day 3–4 at which point they lost track and tried taking a 4 mg dose, which triggered withdrawal.

## Discussion

This study provides insights on experiences of withdrawal from the largest reported retrospective sample to-date of people using fentanyl who underwent buprenorphine LDI as outpatients. Many existing studies of LDI protocols are case reports, case series, or retrospective cohort studies with small sample sizes, and few have been in the outpatient setting. This is especially true of studies of patients primarily using fentanyl. Dosing regimens, patient characteristics, and opioids of use vary among these cohorts as well [10, 12].

Nearly a third of our cohort experienced withdrawal, but most cases were mild. Still, rates of moderate and severe symptoms were higher than in other LDI studies. For example, in the outpatient setting, one small study of 14 patients undergoing LDI had 3 cases of withdrawal symptoms (21%), all of which were mild [28]. A second study of 12 outpatients found mild withdrawal in only one patient (8%) [26]. Similarly, in an inpatient study of 42 people using fentanyl who underwent low dose IV buprenorphine initiation, withdrawal was generally mild as well [32]. We suspect our relatively high rate of withdrawal (31%) and presence of moderate and severe cases may be due to our larger sample size and retrospective design capturing a wider array of experiences of LDI. In this larger sample, more patients may face difficulty with initiation, have needs or preferences that are not as well-suited to LDI, and may have varied opioid use practices including varying amounts of fentanyl use, all of which could impact withdrawal risk.

Precipitated withdrawal was uncommon in our cohort, representing only 8% of 118 initiations with follow up data, and only 3% when considering precipitated withdrawal that occurred despite patients following protocol instructions. We found three papers that discussed precipitated withdrawal in LDI attempts among outpatients using fentanyl, all with sample sizes less than 10 and mostly from Canada [33–35]. Our results showed a higher percentage of initiation attempts with precipitated withdrawal than in these studies, however these studies did not explicitly describe how they defined precipitated withdrawal, making comparison difficult. Additionally, our patients were not on prescribed full-agonist opioid therapy as were some patients in the literature [33, 34]. For instance, an outpatient case series from Vancouver found no precipitated withdrawal in any of 7 patients using fentanyl, though more than half of these patients were continued on methadone or slow-release oral morphine therapy during initiation [33]. Thus, our higher rate of precipitated withdrawal may be because we excluded patients who were on methadone maintenance therapy, and while continued use of non-prescribed full-agonist opioids (e.g. fentanyl) is advised by our clinicians during buprenorphine uptitration, concurrent prescription of controlled full-agonist opioids other than methadone for OUD is not possible due to U.S. law. Additionally, the majority of reports in the literature describe successful LDIs without precipitated withdrawal, suggesting a bias toward reporting successful cases [10].

Given differences in study design and in the definitions of precipitated withdrawal used, it is difficult to compare our findings to results of studies of traditional initiations among people who use fentanyl. While it did not report on precipitated withdrawal specifically, a large retrospective study (*N* = 1679) using a self-report survey of people undergoing traditional initiations found 22% of patients using fentanyl reported severe withdrawal less than 24 hours after last fentanyl use, while 8% did less than 48 h after last use [23]. The recent finding of 0.76% of patients with precipitated withdrawal among emergency department patients undergoing initiation with sublingual or injectable buprenorphine (*N* = 1200) is quite low. This study used a randomized control trial design making its relatively high-quality results convincing [24]. However, its results are difficult to compare to our findings as the emergency department is a very different clinical setting from outpatient buprenorphine initiation, with access to intravenous medication to manage withdrawal and a higher level of care [24]. In contrast to that study, a recent retrospective cohort study in the emergency department (*N* = 347) found a precipitated withdrawal rate of 14% in patients undergoing initiation with sublingual buprenorphine [36].

Steps are taken in our clinics to simplify the initiation process including use of pre-prepared blister packs, experienced clinicians and pharmacists, and close follow up [10, 28]. However, there was still known deviation from protocol instructions in a large percentage of our initiation attempts, including 7 of the 10 cases of precipitated withdrawal. Similarly, in a case series from the emergency department, deviation from guideline-based buprenorphine administration was present in 4 of 13 (31%) traditional initiations with suspected precipitated withdrawal, suggesting that protocol deviation may play a role in withdrawal risk [22]. Following protocol instructions may be challenging in outpatient LDIs, which have complicated, frequent dosing schedules using small, split tablets or films of buprenorphine [10, 26]. This may be especially difficult in the setting of structural barriers, such as homelessness or lack of financial resources, which reduce access to supports like reliable timekeeping devices and places to store medications.

Our sample includes a large number of patients who face significant structural challenges, which contribute to disparities in rates of OUD and overdose. These structural factors, including the effects of systemic racism, can negatively impact patients’ experiences of OUD care as well as their ability to engage in and adhere to care [5, 37]. While some interventions, including those described above with regard to protocol deviation, were used to address structural barriers in our cohort, significant investments in policy-level strategies to address the structural determinants of health will be necessary to reduce the impacts of OUD and opioid overdose on communities that experience ongoing and historical oppression.

Strengths of our study include the large size of our cohort and assessment of chart documentation on withdrawal symptoms by multiple addiction medicine experts. Limitations include our retrospective design and data collection via chart review, which may be subject to biases in documentation. As loss to follow up was high at 33%, and 19% of those with follow up data lacked sufficient documentation to assess withdrawal, the completeness of our data may be limited. Since experiences of withdrawal may impact likelihood of returning for care, this loss to follow up could bias results towards less incidence of withdrawal. The high rate of loss to follow up and lack of documentation make our results difficult to compare to inpatient studies in which patients are more closely monitored. We relied on clinician judgment to define precipitated withdrawal, which may also be open to bias. Due to sample size limitations, we were unable to compare outcomes across groups. We additionally do not have data on use of ancillary medications or their effect on withdrawal symptoms.

## Conclusion

Withdrawal was relatively common in this large cohort of outpatients using daily fentanyl who underwent buprenorphine LDI, but the majority of withdrawal symptoms were mild. Precipitated withdrawal was rare, and most precipitated withdrawal was in cases involving deviation from the prescribed protocol. These data conflict with prior case report studies of LDI citing no withdrawal experienced during initiation. It is possible that when LDI is provided at a larger scale, more patients may experience mild withdrawal symptoms and some may experience precipitated withdrawal. Clinicians implementing LDI for patients using fentanyl should therefore consider counseling patients that they may experience mild discomfort during initiation, and that there is still some risk of moderate to severe symptoms as well as precipitated withdrawal. To address patients’ concerns regarding precipitated withdrawal in the setting of fentanyl use, future studies should prospectively assess precipitated withdrawal and withdrawal severity across low dose and traditional buprenorphine initiation protocols. Future research should examine possible predictors of precipitated withdrawal including co-use of stimulants, which was extremely common in our sample. Additionally, qualitative studies are needed to explore patients’ experiences of withdrawal during LDI, assess barriers to following prescribed initiation instructions, and generate new strategies that may be more manageable or preferable for patients in the face of structural challenges and changing fentanyl use characteristics. This study contributes much-needed data on withdrawal from a large retrospective cohort, which will help guide patient and clinician decision-making regarding optimal strategies for outpatient buprenorphine initiation in the setting of fentanyl use.

### Electronic supplementary material

Below is the link to the electronic supplementary material.

**Supplementary Material 1**: **Supplemental Table 1**. Frequency of symptoms experienced by patients with any withdrawal, by protocol

**Supplementary Material 2**: **Supplemental Table 2**. Adjunctive medications prescribed by protocol.

## Data Availability

The datatsets generated and/or analyzed during the current study are not publicly available due to their sensitive nature, as they contain protected health information and information regarding substance use treatment. De-identified data may be available from the corresponding author upon reasonable request. Data are securely stored at the University of California, San Francisco.

## Abbreviations

LDI: Low dose initiation
OUD: Opioid use disorder
SFDPH: San Francisco Department of Public Health
UCSF: University of California, San Francisco
4-day: 4-day rapid low dose initiation protocol
7-day: 7-day standard low-dose initiation protocol
