# Supplementary material for: Withdrawal during outpatient low dose buprenorphine initiation in people who use fentanyl: a retrospective cohort study
Source: Harm Reduct J. 2024 Apr 9;21:80. doi: 10.1186/s12954-024-00998-9 (PMC11005253; doi:10.1186/s12954-024-00998-9)
Supplement: Supplementary file 1 — Supplementary Material 1: Supplemental Table 1. Frequency of symptoms experienced by patients with any withdrawal, by protocol [file 12954_2024_998_MOESM1_ESM.docx]

**Supplemental Table 1. Frequency of symptoms experienced by patients with any withdrawal, by protocol.**

|  | **Total** | **4-Day** | **7-Day** |
| --- | --- | --- | --- |
|  | **N=37** | **N=17** | **N=20** |
| **Withdrawal symptom** |  |  |  |
| Restlessness | 13 (35%) | 8 (47%) | 5 (25%) |
| Anxiety | 12 (32%) | 5 (29%) | 7 (35%) |
| Nausea | 10 (27%) | 5 (29%) | 5 (25%) |
| Runny nose | 9 (24%) | 5 (29%) | 4 (20%) |
| Perspiration | 8 (22%) | 5 (29%) | 3 (15%) |
| Stomach cramps | 8 (22%) | 2 (12%) | 6 (30%) |
| Body or muscle aches | 7 (19%) | 3 (18%) | 4 (20%) |
| Cold flushes | 7 (19%) | 3 (18%) | 4 (20%) |
| Diarrhea | 6 (16%) | 4 (24%) | 2 (10%) |
| Agitation | 5 (14%) | 2 (12%) | 3 (15%) |
| Hot flushes | 5 (14%) | 3 (18%) | 2 (10%) |
| Tearing | 2 (5%) | 2 (12%) | 0 (0%) |
| Vomiting | 2 (5%) | 1 (6%) | 1 (5%) |
| Yawning | 2 (5%) | 1 (6%) | 1 (5%) |
| Goosebumps | 1 (3%) | 1 (6%) | 0 (0%) |
