# Supplementary material for: Withdrawal during outpatient low dose buprenorphine initiation in people who use fentanyl: a retrospective cohort study
Source: Harm Reduct J. 2024 Apr 9;21:80. doi: 10.1186/s12954-024-00998-9 (PMC11005253; doi:10.1186/s12954-024-00998-9)
Supplement: Supplementary file 2 — Supplementary Material 2: Supplemental Table 2. Adjunctive medications prescribed by protocol. [file 12954_2024_998_MOESM2_ESM.docx]

**Supplemental Table 2. Adjunctive medications prescribed, by protocol.**

|  | **Total** | **4-Day** | **7-Day** |
| --- | --- | --- | --- |
|  | **N=118** | **N=54** | **N=64** |
| **Adjunctive medication** |  |  |  |
| Clonidine | 41 (35%) | 22 (41%) | 19 (30%) |
| Antihistamine (e.g. diphenhydramine, hydroxyzine) | 40 (34%) | 21 (39%) | 19 (30%) |
| Ondansetron | 35 (30%) | 22 (41%) | 13 (20%) |
| Loperamide | 30 (25%) | 20 (37%) | 10 (16%) |
| Gabapentin | 5 (4%) | 3 (6%) | 2 (3%) |
| Over-the-counter analgesic (e.g. ibuprofen) | 2 (2%) | 2 (4%) | 0 (0%) |
| Trazodone | 1 (1%) | 1 (2%) | 0 (0%) |
| Benzodiazepines | 0 (0%) | 0 (0%) | 0 (0%) |
